# Clinical validation study of the Persian version of the Rapid Eye Movement Sleep Behavior Disorder Screening Questionnaire (RBDSQ-PER)

**DOI:** 10.1055/s-0045-1814369

**Published:** 2025-12-22

**Authors:** Mehrdad Sadri, Alia Shakiba, Hamed Amirifard, Vajiheh Aghamollaii

**Affiliations:** 1 Tehran University of Medical Sciences, School of Medicine, Department of Psychiatry, Tehran Tehran Province, Iran.; 2 Northwell Health, Zucker Hillside Hospital, Division of Psychiatry Research, Glen Oaks NY, USA.; 3 Tehran University of Medical Sciences, Iranian Center of Neurological Research, Tehran Tehran Province, Iran.; 4 Tehran University of Medical Sciences, School of Medicine, Department of Neurology, Tehran Tehran Province, Iran.

**Keywords:** REM Sleep Behavior Disorder, Parkinson Disease, Sleep Wake Disorders

## Abstract

**Background:**

Rapid eye movement (REM) sleep behavior disorder (RBD) is marked by abnormal behaviors during REM sleep and is associated with neurodegenerative diseases, such as Parkinson's disease. Early diagnosis is critical for managing these associations effectively.

**Objective:**

To validate the Persian version of the RBD Screening Questionnaire (RBDSQ-PER) for Persian-speaking patients.

**Methods:**

The study involved 171 participants from sleep centers associated with the Tehran University of Medical Sciences, including Parkinson's disease patients with RBD, individuals with obstructive sleep apnea, and healthy controls. The RBDSQ was translated into Persian following established linguistic validation protocols. Reliability and diagnostic utility were measured with Cronbach's alpha to determine internal consistency and the intraclass correlation coefficient.

**Results:**

The RBDSQ-PER demonstrated a Cronbach's alpha and intraclass correlation coefficient of 0.847, indicating strong internal consistency. The analysis of the receiver operating characteristic curve established a cut-off score of 5.5, differentiating individuals with and without RBD with 100% sensitivity and 93% specificity.

**Conclusion:**

The RBDSQ-PER is a reliable tool for screening in Persian-speaking populations, enhancing initial sleep assessments and guiding further diagnostic evaluations. Future research should consider broader patient groups to extend the questionnaire's applicability.

## INTRODUCTION

Rapid eye movement (REM) sleep behavior disorder (RBD) is a parasomnia characterized by abnormal skeletal muscle activity. Clinically, it manifests as motor behaviors and/or vocalizations during REM sleep, a phase typically marked by muscle atonia. These behaviors often align with dream content ranging from speaking and shouting to complex gestures and aggressive movements of the patient's body. 1 2

The prevalence of RBD is estimated to be 0.5 to 1.25 percent in the general population and approximately 2 percent in the elderly. 3 4 Idiopathic or isolated RBD (iRBD), is recognized as one of the strongest risk factors for future occurrence of α-synucleinopathies, including Parkinson's disease (PD), multiple system atrophy (MSA), and dementia with Lewy bodies (DLB). Therefore, early diagnosis of RBD is essential for patients and clinicians to proactively address potential neurodegenerative outcomes. 5 6

A history of recurrent dream-enactment behaviors, reported by patients or their bed partners, warrants further evaluation to confirm the diagnosis of RBD. Video polysomnography (PSG) is the gold standard diagnostic tool, requiring the observation of motor activity during REM cycles, regardless of whether RBD-related behaviors occur during the recording. 7 Additionally, this exam is the most reliable measure to rule out other sleep disorders with similar symptoms, such as obstructive sleep apnea (OSA). 8

However, PSG is time-consuming, expensive, and not widely accessible, making it impractical for use in all individuals suspected of having RBD. To overcome these challenges, various rating scales and clinical methods have been developed to provide low-cost tools for screening and evaluating this condition. Examples of these measures include the RBD Screening Questionnaire (RBDSQ), REM Sleep Behavior Disorder Questionnaire Hong Kong (RBDQ-HK), Mayo Sleep Questionnaire (MSQ), Innsbruck RBD Inventory (RBD-I), REM Sleep Behavior Disorder Single-Question (RBD1Q), and the Movement Disorders Non-Motor Symptom Scale (NMSS). 9 10 11 12 13 14 15 16

The RBDSQ is a self-rating questionnaire developed and validated by Stiasny-Kolster et al. in 2007 in both German and English 11 It assesses the content and frequency of dreams, dream-enactment behaviors/vocalizations, sleep disturbances, and a history of neurological diseases, consisting of 13 items with yes/no answers. 11 This instrument has been translated and validated in several languages including Italian, Czech, Brazilian Portuguese, Japanese, Chinese, Korean, and Turkish. 17 18 19 20 21 22 23 24

To the best of our knowledge, RBDSQ has not yet been studied in the Persian-speaking population. Therefore, the aim of this study was to validate the Persian translation of the RBDSQ (RBDSQ-PER) and assess its reliability and diagnostic value in native speakers.

## METHODS

### Participants

This validation study was designed to evaluate the reliability of the RBDSQ-PER in native speakers. Through convenience sampling, a total of 171 individuals were recruited from two sleep centers affiliated with the Tehran University of Medical Sciences in Tehran, Iran.

The recruitment was conducted between October 2020 and April 2022 leveraging the frequent referral of patients with PD and OSA to these medical centers. The sample consisted of three groups. First was the RBD group, which included 57 participants all diagnosed with PD according to the clinical diagnostic criteria of the United Kingdom Parkinson's Disease Society Brain Bank (UKPDSBB). 25 All of these patients were clinically assessed by an expert neurologist and the diagnosis of RBD was confirmed by PSG following the American Academy of Sleep Medicine's clinical practice guidelines. 26 Other sleep disorders were ruled out in these patients.

Secondly, the OSA group consisted of 57 participants diagnosed with OSA with intact REM stage of sleep and no comorbid sleep disorders. The diagnosis was made by video PSG based on the American Academy of Sleep Medicine clinical practice guidelines. 26 Other sleep disorders were ruled out, and all of the subjects in this group were recruited before the administration of continuous positive airway pressure (CPAP) therapy.

The last group consisted of 57 healthy individuals without any complaints or history of sleep disorders. The members of this group were all selected from the companions of patients from the other two study groups, who were visited by a general practitioner under the supervision of a neurologist to ensure their negative history of neurological and sleep disorders through a semi-structured clinical interview. The video PSG was not performed on this group.

All participants were evaluated to ensure they were not using commonly prescribed psychoactive medications. Informed consent was obtained from all of the subjects. This study was conducted according to the World Medical Association's (WMA) Declaration of Helsinki. 27 Ethical approval was obtained from the Research Ethical Committee of the Tehran University of Medical Sciences before its beginning (no. 13991128).

### Questionnaire translation

Following correspondence with Dr. Karin Stiasny-Kolster, the original author of RBDSQ, and the Mapi Research Trust as the copyright holder, permission was obtained to translate the original English version of the questionnaire into Persian. According to the Linguistic Validation Guidance of a Clinical Outcome Assessment (COA) provided by Mapi, this process included two independent forward translations, reconciliation into a single version, backward translation by a bilingual native English/Persian speaker, cognitive debriefing interviews with patients to assess clarity and cultural relevance, and final proofreading. 28 Details on the translation process and cultural adaptation have been thoroughly described and published in another article. 29

### Clinical evaluation

All the participants completed the RBDSQ-PER. Candidates for the sleep study filled out the questionnaire immediately before or after PSG and before starting treatment. Participants were encouraged to seek input from their bed partner or a close relative when completing the RBDSQ.

To evaluate the test–retest reliability, 41 subjects were randomly selected to complete the questionnaire for the second time after 1 to 3 months. The timing for the second completion of the questionnaire was based on patients' availability. For internal consistency analysis, all baseline questionnaires were included. For test–retest reliability, we included only participants who reported no initiation of treatment for their sleep disorder between the two administrations, to avoid confounding effects on their scores.

Type I overnight PSG was performed in the sleep laboratory with continuous technician supervision, using a standard acquisition system. Synchronized digital audio and infrared video were recorded throughout the night. Several other exams were recorded, including electroencephalography (EEG) of the C3-A2, C4-A1, F3-A2, F4-A1, O1-A2, and O2-A1; bilateral electrooculography (EOG); electrocardiogram (ECG); and five surface electromyography (EMG) leads, including submental, bilateral tibialis anterior, and bilateral flexor digitorum superficialis.

Respiratory monitoring included oronasal airflow measured by both thermistor and nasal pressure cannula, oxygen saturation by finger pulse oximetry, and thoracoabdominal effort using inductance plethysmography belts.

Furthermore, body position was recorded with a built-in position sensor. Signals were digitized and scored by trained sleep specialists blinded to RBDSQ results. The procedure was conducted according to the American Academy of Sleep Medicine's Manual for the Scoring of Sleep and Associated Events. 30

### Statistical analysis

Sample size calculation was performed using the MedCalc Statistical Software (MedCalc Software Ltd.), version 19.5.3, considering type I error (Alpha, Significance) as 0.05 and type II (Beta, 1-Power) as 0.20. Data management and analysis were performed using the IBM SPSS Statistics for Windows (IBM Corp.), version 27.0.

Descriptive analysis was used to report frequencies in different groups and subgroups. Student's t-test and analysis of variance (ANOVA) were used to compare RBDSQ scores among the study groups. Additionally, the Dunnett T3 and Scheffe post-hoc tests were used if applicable. Pearson's correlation and paired sample t-test were also used for participants who completed the questionnaires twice. The intraclass correlation coefficient (ICC) and Cronbach's alpha were used to assess the internal consistency and reliability of the test.

Sensitivity and specificity were calculated for different cut-off values of the total RBDSQ score using the receiver operating characteristic (ROC) curve. The diagnostic ability of the test was evaluated by area under the curve (AUC). We considered *p* -values < 0.05 as statistically significant. Cronbach's alpha coefficient and ICC values ≥ 0.7 and AUC values ≥ 0.65 were considered acceptable. The values reported included a 95% confidence interval (CI) when applicable.

## RESULTS

The RBD group included 36 males and 21 females. The median age was 66 years, with an interquartile range (IQR) of 60–71 years. The OSA group included 34 males and 23 females with a median age of 45 (IQR: 37–52) years. The healthy group included 24 males and 33 females, with a median age of 34 (IQR: 28–42) years, as shown in Table 1 . The analysis for the reliability of the RBDSQ-PER revealed Cronbach's Alpha as 0.847. This result, as well as the item-by-item analysis, are shown in Table 2 . The intraclass correlation coefficient (ICC) was calculated as 0.847 (0.811–0.879, *p* < 0.001).

**Table 1:** Characteristics of the three study groups

	RBD group (n = 57)	OSA group (n = 57)	Healthy group (n = 57)	Total (n = 171)
Male patients (%)	36 (63.2%)	34 (59.6%)	24 (42.1%)	94
Female patients (%)	21 (36.8%)	23 (40.4%)	33 (57.9%)	77
Median age (IQR)	66 (60–71)	45 (37–52)	34 (28–42)	48 (35–63)
Mean duration of PD (years)	5.35 ± 3.46	−	−	5.35 ± 3.46
Median H-Y stage (IQR)	1.5 (1–2.5)	−	−	1.5 (1–2.5)

Abbreviations: H-Y, Hoehn–Yahr scale; IQR, interquartile range; OSA, obstructive sleep apnea; PD, Parkinson's disease; RBD, rapid eye movement sleep behavior disorder.

**Table 2:** Analysis of reliability and diagnostic ability of RBDSQ-PER items

RBDSQ items	1	2	3	4	5	6.1	6.2	6.3	6.4	7	8	9	10
**Cronbach's alpha if item was deleted**	0.845	0.823	0.815	0.825	0.850	0.841	0.824	0.843	0.853	0.844	0.845	0.830	0.825
**Item-total Pearson correlation**	0.478	0.758	0.839	0.735	0.335	0.546	0.758	0.478	0.228	0.494	0.504	0.681	0.731
***p*** **-value**	< 0.001	< 0.001	< 0.001	< 0.001	< 0.001	< 0.001	< 0.001	< 0.001	0.003	< 0.001	< 0.001	< 0.001	< 0.001
**Sensitivity**	0.965	0.965	0.982	0.842	0.193	0.754	0.614	0.298	0.105	0.509	0.877	0.930	1.000
**Specificity**	0.447	0.860	0.956	0.877	0.912	0.605	0.991	0.912	0.904	0.763	0.579	0.737	0.781
**AUC**	0.706 (0.630–0.783)	0.912 (0.865–0.959)	0.969 (0.940–0.999)	0.860 (0.795–0.925)	0.553 (0.459–0.646)	0.680 (0.596–0.764)	0.803 (0.721-0.884)	0.605 (0.511–0.699)	0.504 (0.412–0.597)	0.636 (0.545–0.727)	0.728 (0.651–0.805)	0.833 (0.770–0.896)	0.890 (0.842–0.939)
***p*** **-value**	< 0.001	< 0.001	< 0.001	< 0.001	0.272	< 0.001	< 0.001	0.028	0.926	0.003	< 0.001	< 0.001	< 0.001
**Overall model quality**	0.84	0.87	0.94	0.79	0.46	0.60	0.72	0.51	0.41	0.55	0.65	0.77	0.63

Abbreviations: AUC, area under the curve; RBDSQ-PER, Persian version of the Rapid Eye Movement Sleep Behavior Disorder Screening Questionnaire.

**Table 3:** Sensitivity and specificity of the RBDSQ-PER according to different cut-off values of the total score

Cut-off value	0.50	1.50	2.50	3.50	4.50	5.50	6.50	7.50	8.50	9.50	10.50	11.50	12.50
**Sensitivity**	1.000	1.000	1.000	1.000	1.000	1.000	0.947	0.789	0.614	0.368	0.211	0.088	0.018
**Specificity**	0.079	0.281	0.500	0.737	0.868	0.930	0.947	0.982	1.000	1.000	1.000	1.000	1.000

Abbreviation: RBDSQ-PER, Persian version of the Rapid Eye Movement Sleep Behavior Disorder Screening Questionnaire.

**Figure 1:**
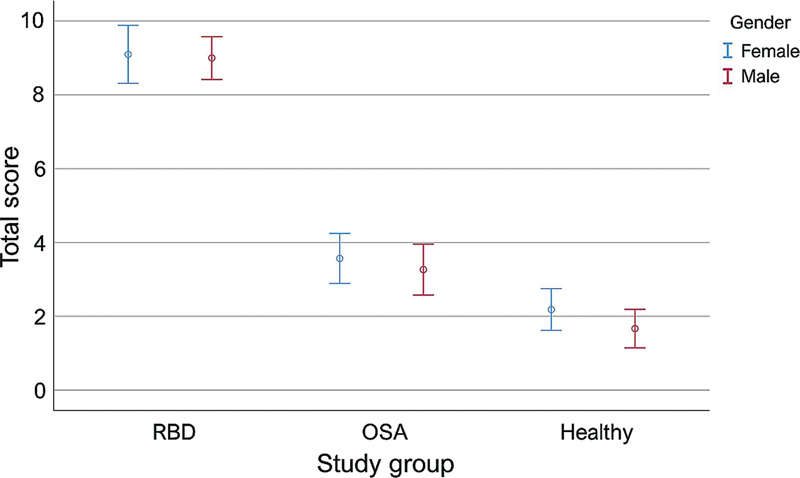
Comparing the means of the total scores on the Rapid Eye Movement Sleep Behavior Disorder (RBD) Screening Questionnaire (RBDSQ) in the study groups. The total score in the RBD patients was significantly higher than that of obstructive sleep apnea (OSA) patients (RBD: 9.04, 8.58–9.49; OSA: 3.39, 2.91–3.87; *p* < 0.001), and healthy individuals (1.96, 1.58–2.35; *p* < 0.001).

The mean total score of the questionnaire with a 95%CI in the RBD, OSA, and healthy groups were 9.04 (8.58–9.49), 3.39 (2.91–3.87), and 1.96 (1.58–2.35), respectively. A comparison of the study groups showed that the mean total scores were significantly different between any groups according to the ANOVA and Dunnett T3 tests ( *p* < 0.001). However, males and females had relatively the same mean total scores within each group ( Figure 1 ).

Considering both OSA and healthy groups as a control non-RBD group, the mean total score of this group with a 95%CI for the mean was 2.68 (2.35–3.01) which is significantly lower than that of the RBD group ( *p* < 0.001).

**Figure 2:**
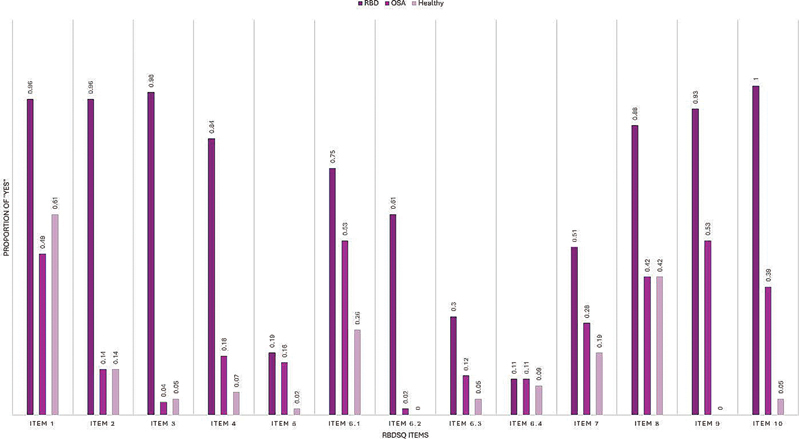
The proportions of “yes” answers to different items of the questionnaire among the study groups.

Item-by-item analysis of the positive answers in different groups showed significantly higher “yes” answers by RBD patients for all of the questions ( *p* < 0.001) except for items 5, 6.3, and 6.4. For items 5 and 6.3, RBD patients had more “yes” answers than the healthy group ( *p* = 0.007 and 0.002 respectively). However, the same comparison between the RBD and OSA groups showed no difference ( *p* = 0.947 and 0.063 respectively). Furthermore, the answers for item 6.4 were not significantly different between any of the groups ( *p* = 0.938). Figure 2 represents the proportions of “yes” answers to different items of the questionnaire among the study groups.

Analysis of the test–retest reliability of the questionnaire through Pearson's correlation test revealed that the total RBDSQ scores the first and second time were significantly correlated ( *p* < 0.001; r = 0.976), though they were not significantly different ( *p* = 0.675).

In the RBD group, the mean duration of the PD was of 5.35 ± 3.46 years. However, PD duration and the total RBDSQ score were not significantly correlated in this study ( *p* = 0.549).

Utilizing the ROC curve analysis to evaluate the diagnostic ability of the RBDSQ-PER, maximum sensitivity of 100% (93.6–100%) and specificity of 93% (86.8–96.6%) were obtained by considering 5.5 as the optimum cut-off value for the differentiation of all RBD patients from the rest of the participants ( Table 3 ). However, among the participants that were labeled as probable RBD by RBDSQ in this study (total score ≥ 6), 87.7% of them were truly RBD patients and 12.3% were misdiagnosed from control groups.

**Figure 3:**
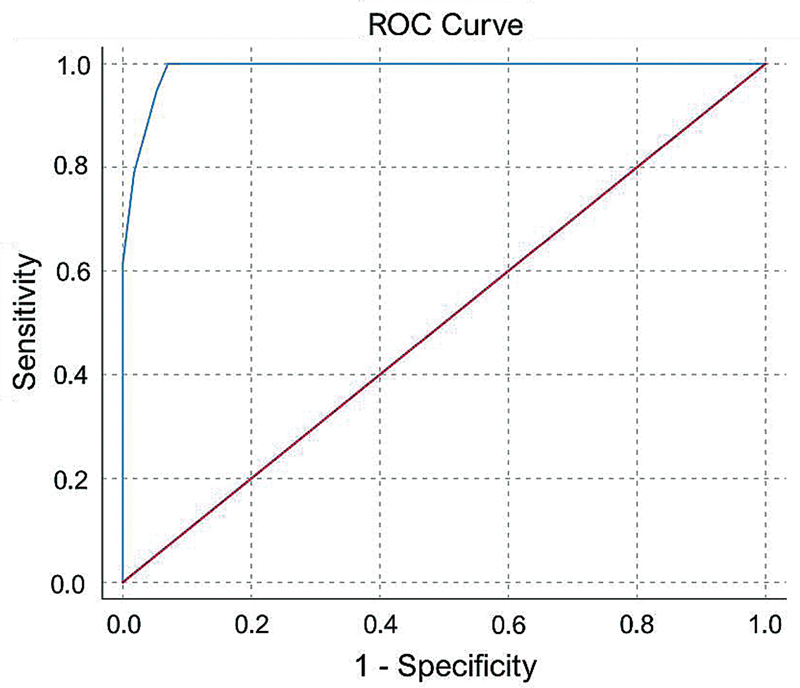
The receiver operating characteristic (ROC) curve's optimum cut-off value was 5.5, as the total RBDSQ score was obtained for the screening of RBD with the sensitivity and specificity of 100 and 93%, respectively (area under the curve = 0.99; 95%CI: 0.98–1.00; *p* < 0.001).

The same cut-off value could distinguish RBD patients from OSA patients with sensitivity and specificity of 100% (93.6–100%) and 87.70% (75.4–94.6%), respectively. Moreover, using this cut-off value for the differentiation of RBD patients from healthy individuals led to sensitivity and specificity of 100% (93.6–100%) and 98.20% (90.7–99.9%), respectively. Additionally, the AUC was calculated as 0.99 (0.98–1.00, *p* < 0.001). The overall model quality based on the total score of the questionnaire was 0.98 ( Table 2 ; Figure 3 ).

## DISCUSSION

Several studies have emphasized the application of a simple, accessible, and inexpensive tool to diagnose RBD. Various instruments ranging from single-question screening tools to more detailed questionnaires have been developed for this reason. The RBDSQ is a widely recognized tool which has been studied in several languages and patient populations. 17 18 19 20 21 22 23 24 The present study was designed to investigate the reliability and validity of the RBDSQ-PER.

This study found that this questionnaire is acceptably reliable, as Cronbach's Alpha was 0.847. The deletion of any of the items did not significantly affect the reliability of the questionnaire in this study as Cronbach's Alpha ranged from 0.815 (without item 3) to 0.853 (without item 6.4), as shown in Table 2 .

Considering the ability of the questionnaire to predict the RBD diagnosis ( Table 2 ), item 3 demonstrated the highest overall model quality (0.94), while item 6.4 showed the lowest (0.41). Item 3 is indicative of the relationship between the movements of the body at night and dreams' content. Answering “yes” to this item probably reflects the main feature of RBD, which is the dream-enactment behaviors at night leading to a better prediction ability. In the current study, RBD patients did not significantly answer more positively to items 5, 6.3, and 6.4 in comparison with the OSA patients and healthy individuals. Therefore, these items had lower diagnostic ability among the items on the questionnaire.

The item-by-item analysis showed that the highest overall sum of sensitivity (98.2%) and specificity (95.6%) was for item 3, with the highest item-test correlation (r = 0.839, *p* < 0.001). Additionally, item 6.4 had the lowest overall sum of sensitivity (10.5%) and specificity (90.4%) with the lowest item-total correlation (r = 0.228; *p* = 0.003). Items 3, 5, 6.2, 6.3, and 6.4 were highly specific for the screening of RBD symptoms. These findings reflect similar results of the original study, in which items 5, 6.3, and 6.4 had the highest specificity in RBDSQ. 11

The RBD group's mean total score in the current study was 9.04 (8.58–9.49). This is in line with that of the original study by Stiasny-Kolster et al., 11 which reported a mean score of 9.5 ± 2.8. However, another study conducted specifically in PD patients reported the mean total score of RBDSQ in RBD patients as 7.5 ± 2.4 in a group with an interview before the administration of the questionnaire and 6.0 ± 3.1 in another group without a prior interview. 31 They concluded that performing an interview before administrating RBDSQ can potentially affect the patient's insight into RBD symptoms and subsequently alter the scores. 31 In our study, a structured interview was not performed before administrating RBDSQ to any participants. Therefore, the evaluation of the possible effects of an interview on the scores is beyond the scope of this study.

An additional limitation is the absence of information on participants' educational level. Because literacy and health-related knowledge can influence both understanding of questionnaire items and accuracy of self-reporting, this missing variable may have affected the RBDSQ-PER performance and should be addressed in future studies.

An important finding of this study was the cut-off value of 5.5 for the RBDSQ-PER score, which resulted in sensitivity and specificity of 100% and 93% respectively. Accordingly, a total score of 6 or higher could distinguish patients with and without RBD with an acceptable diagnostic ability in screening.

However, other studies reflect differences in cut-off values, sensitivity, and specificity. The original study reported a sensitivity of 96% and a specificity of 56%, with a cut-off value of 5. 11 Validation studies in other languages such as Chinese (sensitivity = 92.1%; specificity = 81.2%), Czech (sensitivity = 95.1%; specificity = 59.6%), Japanese (sensitivity = 88.5%; specificity = 90.9–96.9%), and Turkish (sensitivity =100%; specificity = 64%) reported 5 as the optimal cut-off value to differentiate individuals with and without RBD. 18 20 21 22 23

In contrast, the validation of RBDSQ's Italian version by Marelli et al. indicated that the optimal cut-off value was 8 (sensitivity = 82.9%; specificity = 82.0%). 17 The Brazilian validation study found an optimal cut-off score of 4, yielding a sensitivity of 84% and specificity of 57.9%. This slightly lower threshold further illustrates the heterogeneity of optimal cut-off scores across populations. 19

As mentioned before, our study found the cut-off value of 6 as the optimal score to differentiate RBD patients from OSA patients and healthy individuals. The variability in optimal cut-off values—ranging from 4 in the Brazilian study to 8 in the Italian version—likely reflects differences in patient populations (such as idiopathic versus PD-associated RBD), the prevalence of comorbid sleep disorders, severity of RBD symptoms, availability of a bed partner's input, and structured interview preceding questionnaire administration.

These differences highlight that the RBDSQ should be viewed primarily as a screening instrument and not a diagnostic tool. Therefore, clinicians should consider local patient characteristics and the intended balance between sensitivity and specificity when selecting a cut-off for clinical or research use. A possible reason for the discrepancies could be the difference in symptom severity in the RBD study groups in different investigations. Therefore, according to the fluctuating nature of these symptoms, it should be taken into consideration that the result of RBDSQ is uncertain.

Another reason for the difference in results across studies could be their uneven clinical settings. For instance, RBDSQ has been administered following an interview in one study by Stiasny-Kolster et al. 31 Also, patients have been selected from different pathophysiologic backgrounds, such as PD, iRBD, narcolepsy etc. 18 20

The availability and quality of information provided by bed partners could be another factor to consider when interpreting the results of these studies, as the administration of the RBDSQ can also involve input from patients' bed partners. Although participants were encouraged to seek input from their bed partner or a close relative when completing the RBDSQ-PER, we did not systematically record whether a companion actually assisted. Given that the presence of a bed partner is often the main source of information about dream-enactment behaviors, this is another limitation.

The generalizability of our findings is also limited by the composition of the study groups. All RBD patients in our study were recruited from the PD patient population and were receiving standard antiparkinsonian treatment, so possible confounding effects of such treatment cannot be ruled out. We did not include patients with iRBD, narcolepsy-related RBD, epilepsy, or other parasomnias that may present diagnostic challenges for screening questionnaires due to overlapping symptomatology.

Moreover, the healthy group was defined by the absence of self-reported sleep complaints and medical history rather than by PSG confirmation. Therefore, we cannot fully exclude the presence of subclinical or unrecognized sleep disorders among healthy controls. This potential misclassification could have influenced specificity estimates. In contrast, super-healthy controls could have made the test look more specific than it would be in real clinical practice and may not generalize to community populations.

Future studies should include larger and more diverse populations of RBD patients from varied pathological backgrounds, along with multiple control groups, to further validate the use of the RBDSQ as a screening tool and to tune the optimal cut-off value.

In conclusion, this study served as a validation of the RBDSQ-PER. The findings demonstrated that this is a reliable tool for screening symptoms, helping to distinguish RBD patients from those with OSA or healthy individuals. The RBDSQ-PER can be valuable in the initial sleep assessment, guiding further planning for advanced sleep studies in patients presenting with these symptoms.
